# Food insecurity among displaced populations in Armenia during the 2020 Nagorno-Karabakh conflict

**DOI:** 10.3389/fpubh.2024.1499523

**Published:** 2024-12-11

**Authors:** Araz Majnoonian, Carine Tamamian, Musheh Ovanesian, Tala Al-Rousan

**Affiliations:** ^1^Herbert Wertheim School of Public Health and Human Longevity Science, University of California, San Diego, La Jolla, CA, United States; ^2^School of Public Health, College of Health and Human Services, San Diego State University, San Diego, CA, United States; ^3^School of Medicine, University of California, San Diego, La Jolla, CA, United States; ^4^University of California, San Diego, La Jolla, CA, United States

**Keywords:** displacement, food security, food insecurity, humanitarian, war conflict, Armenia, Nagorno-Karabakh conflict, Nagorno Karabakh

## Abstract

**Background:**

The 2020 Nagorno-Karabakh conflict resulted in displacement of approximately 90,000 ethnic Armenians from Nagorno-Karabakh to Armenia, exacerbating existing vulnerabilities in the region. This study investigated food insecurity among displaced populations and host communities in Armenia during the conflict.

**Methods:**

This study is a secondary analysis of cross-sectional data obtained from the 2020 REACH ARM Database Multi-Sector Needs Assessment (MSNA), which was conducted across six Armenian provinces. The original data collection was designed to assess humanitarian needs in Armenia in the aftermath of the 2020 Nagorno-Karabakh conflict. In this analysis, we examined the associations between displacement status and two outcomes of interest: the household’s ability to purchase food and reduced portion sizes. Multivariable logistic regressions were performed for each food security outcome.

**Results:**

The study sample included 1,309 households, with most male heads (68.1%), an age distribution mostly under 50 years (81.2%), a majority having general education (55.7%) and higher education (22.8%), and 74.0% not employed. 134 households (10.2%) were displaced and staying in collective centers, 658 households (50.3%) were displaced and staying with family or friends, and 517 households (39.5%) that were not displaced but hosting displaced people. Displaced individuals staying in collective centers had 3.89 times higher odds of reporting a reduced ability to purchase food compared to non-displaced individuals (aOR: 3.89, CI: 2.396.45). Additionally, displaced individuals staying with friends or family had 2.5 times higher odds of experiencing food purchasing difficulties (aOR: 2.53, 95% CI: 1.87–3.42). Households in debt and those with children and lactating women also faced higher food insecurity risks. Similarly, displaced individuals in collective centers had 1.94 times the odds of reducing portion sizes (aOR: 1.94, 95% CI: 1.12–3.29). Female-headed households and households with lactating women also experienced higher rates of portion size reduction, while higher-income households were less likely to face such issues.

**Conclusion:**

Our findings underscore the critical need for targeted humanitarian interventions to address food insecurity among displaced populations in conflict settings. Displaced individuals, especially those in collective centers, faced higher risks of food insecurity, compounded by household vulnerabilities such as debt, children, and lactating women. Female-headed households were particularly affected, necessitating gender-sensitive humanitarian interventions.

## Introduction

Globally, over 120 million people have been forcibly displaced, including 43.4 million refugees and 63.3 million internally displaced individuals (1, 2). The UNHCR reports that as many as 80% of displaced individuals experience food insecurity, a situation that has been exacerbated by the COVID-19 pandemic (3). Food security is defined by consistent access to safe, nutritious, and culturally appropriate food, and is assessed across availability, access, utilization, and stability (3, 4). Displaced individuals often face restrictive laws and policies that hinder their ability to access essential resources, increasing their risk of food insecurity (3).

Certain groups within displaced populations, such as women, children, the older adults, and people with disabilities, face heightened vulnerabilities during displacement. Women and children are at increased risk of violence and exploitation, while the older adults and those with disabilities face barriers to accessing resources and services, heightening their risk of food insecurity. Food insecurity can lead to long-term health issues, including malnutrition in children, which contributes to chronic diseases later in life (3, 5, 6).

The Nagorno-Karabakh (NK) conflict, a long-standing territorial dispute between Armenia and Azerbaijan, escalated dramatically in September 2020, leading to intense military confrontations and significant civilian displacement (7). The predominantly ethnic Armenian enclave, which had a population of approximately 150,000, saw extensive shelling of major cities. The conflict forced approximately 90,000 residents of NK to flee to neighboring Armenian cities and other urban areas within Armenia (8, 9). The REACH initiative conducted a needs assessment in late 2020 to help prioritize humanitarian aid and address the immediate needs of the displaced population and host communities in Armenia (10).

Displacement disrupts access to essential resources and exacerbates food security challenges (6). In low- and middle-income countries, where 75% of refugees are hosted, a majority of refugees live on less than $2 per day, worsening their vulnerability to hunger and malnutrition (11). The influx of displaced populations from NK into Armenia strained local resources and worsened existing food security issues (8, 12). In 2019, 15% of Armenia’s population was food insecure, and the arrival of thousands of refugees from NK further intensified these challenges (13). The World Food Programme (WFP) reported that by 2022, food insecurity rates in Armenia had risen to 30%, with only one in five households classified as food secure (14). The WFP uses three key indicators to measure food security: the Food Consumption Score, the Reduced Coping Strategy Index, and the Livelihood Coping Strategies. Households are considered food secure if they have an acceptable food consumption score, do not resort to coping mechanisms to secure food, and maintain a stable economic situation (14).

Displaced populations face specific challenges, including the loss of livelihoods, limited access to food markets, and dependence on humanitarian aid (7, 12). Host communities also experience increased pressure on their resources, leading to potential tensions and competition for food supplies (6). Humanitarian aid in the context of displacement are a critical component in addressing food security among these displaced populations, providing essential support to mitigate the immediate impacts of the crisis (15). Consequently, understanding food insecurity in the context of displacement is crucial for developing effective humanitarian responses and policies. Food insecurity disproportionately affects vulnerable groups within displaced populations, including children, pregnant and lactating women, and older adults, who are at higher risk of malnutrition and associated health complications (5, 6, 16, 17). Prolonged food insecurity has significant physical and public health implications, potentially leading to nutrient deficiency and developmental and chronic health issues (16).

The objective of this study is to examine the challenges faced by displaced populations in conflict settings and to inform humanitarian interventions to improve food security outcomes. Specifically, this study examines the relationship between displacement status and food insecurity among populations affected by the NK conflict in Armenia, and how demographic factors such as sex, age, and education level may moderate this relationship.

## Methods

### Study design and participants

This study is a secondary analysis utilizing de-identified and anonymized data from the REACH ARM Database Multi-Sector Needs Assessment (MSNA) (10). The original data were collected as part of a cross-sectional humanitarian needs assessment conducted between November 23 and December 21, 2020. The data collection involved phone interviews with the Head of Household or a household representative. Importantly, we were not involved in the original data collection process. Our analysis was based on publicly available data, and permission to access and utilize these data for research purposes was obtained from the REACH organization. All findings reported in this study are derived from this secondary analysis of the publicly available dataset.

### Sampling

According to the Armenian Ministry of Foreign Affairs, between September and.

December 2020, an estimated 90,000 ethnic Armenians were displaced from NK to Armenia (7, 18). The study sample comprised three distinct groups of the population residing in six provinces of Armenia: Syunik, Ararat, Yerevan, Vayots Dzor, Armavir, and Kotayk. The groups included: i. Non-displaced population hosting displaced individuals; ii. Displaced populations housed in collective centers; iii. Displaced populations staying with friends or family. The sampling and data collection process were conducted by the REACH organization as part of a humanitarian needs assessment. Purposive sampling using a 95% confidence interval and 7% margin of error per strata for each population group and each region was employed to select households and capture a representative sample from each group within the specified provinces (10).

For this secondary analysis, households were included if they had complete data on the exposure and outcome variables, and key demographic variables including age, sex, education level, and employment status of the head of the household, household income, debt, and presence of children, pregnant, and lactating women in the household. Missing data were excluded if surveys were incomplete or lacked these key variables.

### Outcomes

We used two proxy measures to assess food security: 1. Self-report of the household’s ability to purchase food, in which respondents were asked, “Did the conflict reduce your household’s ability to purchase food?” (yes/no); and 2. Respondents self-report of their households’ reduction in portion sizes with the following question: “In the past seven days, if there have been times when you did not have enough food or money to buy food, has anyone in your household had to limit portion sizes at meals?” (yes/no).

These two proxy measures were selected from the publicly available dataset. As this was a secondary analysis, we were limited to the questions included in the original dataset. We did not have input into the design of the survey or the choice of the questions. However, used these two measures because they directly assess immediate food security. The World Food Programme (WFP) recognizes coping strategies, such as the inability to purchase food and reducing portion sizes as direct indicators of acute food insecurity in crisis settings, where immediate survival is often the priority (19). These measures provide a straightforward and immediate assessment of food insecurity, reflecting the day-to-day challenges households faced during the crisis.

### Exposures of interest

The key variable of interest was the displacement status of each household. Other exposure variables were grouped into two domains: Demographic Factors and Household Vulnerabilities. Demographic factors include the head of household’s sex, age, education level, current monthly income, debt, and employment status. Household vulnerabilities include the presence of children and pregnant and lactating women in the household.

### Statistical analysis

RStudio was used for data cleaning, tabulation, and analysis. Descriptive statistics for categorical variables are presented as frequencies and percentages, and associations between variables were assessed using Chi-square tests and Fisher’s exact tests, where appropriate. Age was originally a continuous variable but was categorized into discrete age groups (<29, 30–39, 40–49, 50–59, and ≥ 60 years) to enhance interpretability and facilitate comparisons across meaningful demographic groups. This approach also accounts for the skewed distribution of age in the sample, providing a more robust analysis across age categories. Income was a categorical variable in the dataset with predefined income groups, so normality testing and measures such as median and IQR were not applicable to these variables.

We observed the association between displacement status and each potential risk factor of interest. All variables with a statistical association with each outcome with a *p*-value less than 0.15 were included in the multivariable analysis, along with the primary exposure (displacement status) and the demographic variables of sex and age. The threshold of 0.15 was chosen prior to analysis for a purposeful selection process to reduce the chances of incorrectly excluding potentially important risk factors in the multivariable model (20). We performed bivariate and multivariate analyses to examine associations between displacement status and outcomes of interest while controlling for potential confounding variables.

We conducted multivariable logistic regression models for each food security outcome and structured these models around two domains: Demographic Factors and Household Vulnerabilities. Initially, we fitted a base model that included displacement status and the demographic variables of sex and age. We included variables from Domain 1 (Demographic Factors) that were retained from the univariable analysis one at a time based on likelihood ratio tests (LRT) or *p*-values to assess their association with each outcome. We excluded variables that did not lead to a change of at least 10% in the effect estimates of the model or had non-significant LRT results. Variables with well-documented significance in the literature were retained. Following the same process, the revised base models were used as we incorporated variables from the next domain (Domain 2: Household Vulnerabilities).

## Results

The total sample size was 1,324 households. Due to incomplete survey data, 15 households were dropped, resulting in a total sample size of 1,309 households. There were 134 (10.2%) households that were displaced and staying in collective centers, 658 (50.3%) households that were displaced and staying with family or friends, and 517 (39.5%) households that were not displaced but hosting displaced people. The sample’s demographic characteristics are stratified by displacement status and shown in Table 1. Most heads of households were male (68.1%), while most survey respondents were female (76.2%). The mean age of the head of household was 33.3 years old (SD = 14.15) and most had at least completed general education through grade 12 (55.7%). Most households had an average monthly income of <120,000 Armenian Drams (~ $308 USD) and 369 households (28.2%) had no income. Most heads of households reported being not employed (74.0%) and in debt (55.8%). The majority of households had children (70.3%). A total of 89 households had pregnant women (6.8%), and 180 households had lactating women (13.8%). Overall, 63.4% of households reported that the conflict reduced their ability to purchase food in the past 7 days. Among these households, the majority were displaced and staying with family or friends (56.7%). Specifically, 77.6% of households displaced and residing in collective centers reported that the conflict negatively impacted their food purchasing ability during this period. Similarly, 71.6% of those displaced and staying with friends or family experienced a reduction in their ability to purchase food due to the conflict. Additionally, 49.3% of individuals hosting displaced families reported a diminished capacity to purchase food as a result of the conflict. Some respondents (14.3%) reported that their household had eaten reduced portions in the last 7 days. Variables that were significantly associated with each outcome are listed in Table 2.

**Table 1:** Sample socio-demographic characteristics by displacement status.

Variables of Interest	Total	Displacement Status N (%)			*p* value*
	(*n* = 1,309)	HOC	DCC	DSF	
		(*n* = 517)	(*n* = 134)	(*n* = 658)	
Sex of Head of Household					
Male	891 (68.1)	337 (65.2)	84 (62.7)	470 (71.4)	0.028
Female	418 (31.9)	180 (34.8)	50 (37.3)	188 (28.6)	
Age in years of Head of Household					
<29	534 (40.8)	157 (30.4)	61 (45.5)	316 (48.0)	<0.001
30–39	295 (22.5)	128 (24.8)	38 (28.4)	129 (19.6)	
40–49	307 (23.5)	147 (28.4)	22 (16.4)	138 (21.0)	
50–59	126 (9.6)	55 (10.6)	9 (6.7)	62 (9.4)	
> = 60	47 (3.6)	30 (5.8)	4 (3.0)	13 (2.0)	
Education Level of Head of Household					
No school	26 (2.0)	6 (1.2)	1 (0.8)	19 (2.9)	0.294
General Education (grade 1–12)	729 (55.7)	282 (54.6)	81 (60.5)	366 (55.6)	
Vocational Education (2-year degree)	256 (19.6)	107 (20.7)	25 (18.7)	124 (18.8)	
Higher Education (Bachelor’s and beyond)	298 (22.8)	122 (23.6)	27 (20.2)	149 (22.6)	
Current Monthly Household Income (AMD)					
No income, dependent on aid or remittances	369 (28.2)	64 (17.3)	61 (16.5)	244 (66.1)	<0.001
Less than 68,000 (minimum wage)	350 (26.7)	141 (27.3)	38 (28.4)	171 (26.0)	
68,000–185,000 (average salary)	378 (28.9)	181 (35.0)	26 (19.4)	171 (26.0)	
More than 185,000	130 (9.9)	85 (16.4)	4 (3.0)	41 (6.23)	
Do not know/ prefer not to say	82 (6.26)	46 (8.9)	5 (3.7)	31 (4.7)	
Debt					
Yes	730 (55.8)	306 (59.2)	56 (41.8)	368 (55.9)	<0.001
No	579 (55.8)	211 (40.8)	78 (58.2)	290 (44.1)	
Employment Status of Head of Household					
Not employed	969 (74.0)	261 (50.5)	127 (94.8)	581 (88.3)	
Employed	256 (19.6)	222 (43.0)	4 (3.0)	30 (4.6)	<0.001
Community or military service	41 (3.1)	28 (5.4)	0 (0.0)	13 (2.0)	
Other	43 (3.3)	6 (1.2)	3 (2.2)	34 (5.2)	
Household Vulnerabilities					
Children in household					
Yes	920 (70.3)	334 (64.6)	90 (67.2)	496 (75.4)	<0.001
No	389 (29.7)	183 (35.4)	44 (32.8)	162 (24.6)	
Pregnant women					
Yes	89 (6.8)	38 (7.4)	11 (8.2)	40 (6.1)	0.547
No	1,220 (93.2)	479 (92.7)	123 (91.8)	618 (93.9)	
Lactating women					
Yes	180 (13.8)	72 (13.9)	11 (8.2)	97 (14.7)	0.133
No	1,129 (86.3)	445 (86.1)	123 (91.8)	561 (85.3)	

HOC, Not displaced, hosting displaced persons; DCC, Displaced, staying in Collective Center; DSF, Displaced, staying with family/friends. *p value from Pearson’s chi-squared test comparing variable with Displacement Status.

**Table 2:** Covariates found to be significantly associated with each food security outcome in the univariable analysis.

	Reduced ability to purchase food due to conflict	Limit portion sizes in last 7 days
	
Primary Exposure	Displacement status**	Displacement status**
Domain 1: Demographic Factors		Sex of HH**
	Age of HH*	
	Education level of HH**	Education level of HH**
	Current monthly income**	Current monthly income**
	Debt**	Debt**
	Employment status**	Employment status**
Domain 2: Household Vulnerabilities	Children in household**	Children in household*
	Lactating women**	Lactating women**

HH, Head of Household. *p value < 0.15. **p value < 0.05.

In the final adjusted model for reduced ability to purchase food (Table 3), displaced individuals staying in community centers had 3.89 times higher odds of reporting a reduced ability to purchase food than those not displaced (aOR: 3.89, 95% CI: 2.39–6.45, *p* < 0.001). Displaced individuals staying with friends or family had 2.5 times higher odds of reporting a reduced ability to purchase food than those not displaced (aOR: 2.53, 95% CI: 1.87–3.42, *p* < 0.001). Households in debt had 58% higher odds of experiencing difficulties purchasing food due to the conflict than households without debt (aOR: 1.58, 95% CI: 1.24–2.03, *p* < 0.001). The presence of children in the household (aOR: 1.47, 95% CI: 1.12–1.93, *p* = 0.006) and lactating women (aOR: 1.92, 95% CI: 1.33–2.83, *p* = 0.001) were associated with increased odds of reporting reduced ability to purchase food.

**Table 3:** Adjusted logistic regression model for reduced ability to purchase food due to conflict.

	Adjusted OR (95% CI)	*p* value
Displacement Status		
Not Displaced, but hosting displaced family	Ref	
Displaced, Staying in Community Center	3.89 (2.39, 6.45)	<0.001*
Displaced, Staying with Friends or Family	2.53 (1.87, 3.42)	<0.001*
Demographic Factors		
Sex of head of household		
Male	Ref	
Female	1.19 (0.91, 1.55)	0.21
Age of head of household		
<29	Ref	
30–39	0.88 (0.63, 1.22)	0.43
40–49	1.00 (0.71, 1.39)	0.98
50–59	0.97 (0.62, 1.54)	0.90
> = 60	0.70 (0.36, 1.37)	0.30
Education Level of Head of Household		
No school	Ref	
General Education (grade 1–12)	2.70 (1.17, 6.42)	0.021*
Vocational Education (2-year degree)	3.35 (1.41, 8.18)	0.007*
Higher Education (Bachelor’s and beyond)	2.49 (1.06, 6.03)	0.039*
Current Monthly Household Income (AMD)		
No income, dependent on aid or remittances	Ref	
Less than 68,000 (minimum wage)	1.77 (1.26, 2.50)	0.001*
68,000-185,000 (average salary)	1.01 (0.72, 1.41)	0.96
More than 185,000	1.08 (0.67, 1.74)	0.75
Do not know/ prefer not to say	1.06 (0.63, 1.80)	0.82
Debt		
No	Ref	
Yes	1.58 (1.24, 2.03)	<0.001*
Employment Status of Head of Household		
Not employed	Ref	
Employed	0.83 (0.58, 1.20)	0.32
Community or military service	0.71 (0.36, 1.41)	0.33
Other	0.90 (0.45, 1.88)	0.78
Household Vulnerabilities		
Children in household		
No	Ref	
Yes	1.47 (1.12, 1.93)	0.006*
Lactating women		
No	Ref	
Yes	1.92 (1.33, 2.83)	0.001*

OR, Odds Ratio; CI, Confidence Interval. *p-value < 0.05.

In the final adjusted model for a household member eating reduced portion sizes (Table 4), displaced individuals staying in community centers have 1.94 (95% CI: 1.12–3.29, *p* = 0.016) times the odds of eating reduced portions in the last 7 days than those not displaced. Female headed households had approximately 43% greater odds of experiencing a reduction in portions consumed by anyone in the household than male-headed households (aOR: 1.43, 95% CI: 1.022.00, *p* = 0.039). Households with higher incomes had approximately 71% lower odds of experiencing reduced portions consumed by anyone in the household than households with lower incomes (aOR: 0.29, 95% CI: 0.11–0.66, *p* = 0.006). Households with lactating women ha d about 76% higher odds of experiencing reduced portions consumed by anyone in the household than households without lactating women (aOR: 1.76, 95% CI: 1.15–2.65, *p* = 0.008).

**Table 4:** Adjusted logistic regression model for anyone in the household eating reduced portions in the last 7 days.

	Adjusted OR (95% CI)	*p* value
Displacement Status		
Not Displaced, but hosting displaced family (Ref)	1	
Displaced, Staying in Community Center	1.94 (1.12, 3.29)	0.016*
Displaced, Staying with Friends or Family	1.30 (0.89, 1.89)	0.177
Demographic Factors		
Sex of head of household		
Male (Ref)	1	
Female	1.43 (1.02, 2.00)	0.039*
Age of head of household		
<29 (Ref)	1	
30–39	0.87 (0.56, 1.33)	0.518
40–49	0.96 (0.62, 1.47)	0.850
50–59	0.66 (0.33, 1.23)	0.214
> = 60	0.90 (0.32, 2.16)	0.826
Education Level of Head of Household		
No school (Ref)	1	
General Education (grade 1–12)	1.48 (0.49, 6.41)	0.534
Vocational Education (2-year degree)	1.59 (0.51, 7.04)	0.471
Higher Education (Bachelor’s and beyond)	0.74 (0.23, 3.31)	0.644
Current Monthly Household Income (AMD)		
No income, dependent on aid or remittances	1	
Less than 68,000 (minimum wage)	0.82 (0.55, 1.23)	0.344
68,000–185,000 (average salary)	0.65 (0.42, 1.00)	0.050*
More than 185,000	0.29 (0.11, 0.66)	0.006*
Do not know/ prefer not to say	1.02 (0.50, 1.95)	0.957
Debt		
No (Ref)	1	
Yes	1.50 (1.07, 2.11)	0.019*
Household Vulnerabilities		
Children in household		
No (Ref)	1	
Yes	1.24 (0.85, 1.83)	0.279
Lactating women		
No (Ref)	1	
Yes	1.76 (1.15, 2.65)	0.008*

OR, Odds Ratio; CI, Confidence Interval. *p-value < 0.05.

## Discussion

The results of our study highlight how sudden and large-scale displacement exacerbates food insecurity among displaced populations. The higher prevalence of reduced food purchasing ability in collective centers (77.6%) and among those staying with family or friends (71.6%) compared to non-displaced host households (49.3%) indicates that displacement status is a critical determinant of food security. These findings align with existing literature on the relationship between conflict, displacement, and food insecurity. Collectively, this work underscores the significance of violent conflict as a major driver of food insecurity (21) and suggests displacement due to conflict further disrupts access to essential resources, leading to heightened food insecurity among displaced populations (22).

While all displaced participants experienced higher odds of food insecurity, those residing in collective centers faced greater challenges in securing adequate food compared to those staying with family and friends. Accordingly, having a social network and the support of family and friends may play a crucial role in mitigating food insecurity among displaced individuals. Social networks can ease access to resources such as food, money, and information about social services, which can mitigate the effects of food insecurity. The role of social networks in building collective resilience among communities has been widely studied in humanitarian crisis settings (23, 24). Social networks provide informal support systems, where community members assist each other with food, shelter, and emotional support (24, 25). These mutual aid networks can quickly mobilize resources and provide immediate relief for the displaced population, filling gaps that humanitarian aid may not cover. Strengthening these networks in humanitarian settings and integrating them into humanitarian response strategies can enhance the effectiveness of interventions.

Households with children and lactating women were more vulnerable to food insecurity. These results are consistent with research highlighting the disproportionate impact of food insecurity on vulnerable groups within displaced populations (5, 17, 22, 26, 40). During periods of displacement, these groups face compounded challenges due to disrupted access to healthcare, school, reduced household income, and the breakdown of social support networks (5). Studies from various conflict zones have documented that households with children and lactating women are often forced to adopt extreme coping strategies, such as reducing meal sizes or skipping meals altogether. These coping mechanisms can have detrimental effects on the health and well-being of both mothers and children, further entrenching the cycle of poverty and food insecurity (5, 22, 26).

The reduction in portion sizes among female-headed households reflects gendered dimensions of food insecurity, often reported in humanitarian contexts (17, 27). Female-headed households are particularly vulnerable due to a range of factors including lower average incomes, limited access to employment opportunities, and greater caregiving responsibilities (17, 28, 29). These factors can force women to adopt coping strategies such as reducing meal sizes or skipping meals to ensure that their children are fed, highlighting the unequal burden of food insecurity on women (3, 17, 30). In conflict settings, the absence of men as they participate on the frontlines impacts household dynamics, leaving women to assume sole responsibility for providing and caring for their families under extremely challenging circumstances (28, 31). This scenario often exacerbates the economic and social vulnerabilities faced by women. Research has shown that women in conflict settings frequently face additional barriers such as gender-based violence, social isolation, and lack of access to social safety nets, all of which can exacerbate their food insecurity (17, 31). Further investigation of the nature of household leadership during conflicts can help in designing targeted interventions that effectively support both temporary and permanent female-headed households. Additionally, providing psychosocial support to women who are managing households alone can help alleviate the emotional and mental strain associated with their roles during times of conflict.

These findings are significant given recent developments in the region. NK has a complex and contested history that has impacted the lives and stability of its inhabitants. Following the collapse of the Soviet Union in the 1990s, NK became a disputed territory between Armenia and Azerbaijan, leading to prolonged conflict and displacement (8, 9, 32–35). This enduring conflict has resulted in repeated waves of displacement and ongoing humanitarian crises, with severe impacts on food security and access to essential resources (12, 32–35). The historical backdrop of territorial disputes and ethnic tensions has shaped the current crisis, contributing to an environment where displaced Armenian populations face heightened vulnerabilities, particularly in terms of food insecurity (12, 32, 33). In October 2023, Azerbaijan seized control and occupied the entirety of NK, forcibly displacing its indigenous Armenian population. This was preceded by a 9-month blockade that had left the population of Artsakh starving (9). Artsakh’s entire population of 150,000 was forcefully displaced to Armenia, now seeking permanent settlement in Armenia (9, 36). Given the current humanitarian crisis of mass displacement and recognizing the importance of social networks, the government of Armenia and humanitarian organizations on the ground may use this evidence to focus their efforts on keeping Artsakh’s displaced communities intact as they permanently settle in Armenia. Future research should be conducted to determine the impact of the blockade and permanent forced displacement on food security outcomes and nutrient deficiency in the refugee population. Further, studies examining social networks as a potential protective factor against food insecurity should be conducted to identify effective support mechanisms.

The findings of this study have significant implications for policy and practice. Firstly, there is a need for targeted food assistance programs that prioritize displaced populations, particularly those in collective centers. Humanitarian aid efforts should prioritize identifying and providing aid to households with children, pregnant, and lactating women to ensure equitable access to nutritious food in households with vulnerabilities. As recommended by the UNHCR and World Health Organization, regular health and nutrition screenings should be implemented at aid distribution sites and community centers during crises (37–39). These screenings can help identify malnourished children, pregnant, and lactating women, providing tailored nutritional support and medical care (37, 38). Future research should investigate the long-term food security outcomes of displaced populations, incorporating longitudinal data to capture changes over time (29). Further, establishing robust monitoring and evaluation systems to track the impact of aid programs on household food security and nutritional outcomes should be considered (32). Continuous data collection and analysis can help refine and improve the targeting of aid efforts (40). Secondly, policies aimed at reducing household debt and improving economic opportunities for displaced populations can mitigate the risk of food insecurity (32). We recommend implementing livelihood support programs that enhance the income-generating capacities of displaced individuals, especially for female-headed households.

### Limitations

Our study’s findings should be considered in light of certain limitations. First, the cross-sectional nature of the data limits the ability to infer causal relationships or temporality between displacement status as an exposure and food insecurity outcomes. Additionally, reliance on self-reported data introduces the potential for recall bias. Regarding our outcome measures, we acknowledge that more validated tools, such as the Food Insecurity Experience Scale (FIES), might provide a more comprehensive assessment of food security, but given the limited publicly available data from the conflict, the chosen proxies offer critical insights into the acute food security challenges during the crisis. Given we were not part of data collection efforts, we can only assume that the urgency and complexity of the post-conflict situation necessitated a focus on these specific, actionable survey questions to address the needs of the displaced population. These questions reflect the immediate coping mechanisms employed by displaced households, which are essential for understanding the short-term impact of the conflict on food access. While these measures are not as comprehensive as a tool like FIES, they were deemed appropriate for the context of this secondary data analysis. Although the use of these proxies may limit the generalizability of the findings to broader food security assessments, they provide valuable, actionable insights into the food security situation of displaced populations in the immediate aftermath of the conflict. Given the constraints of this secondary analysis, we believe these indicators provide valuable, though limited, insights that are highly relevant for emergency response planning. We recommend that future studies consider incorporating FIES or similar tools to allow for more comprehensive and standardized evaluations of food insecurity. This would provide a more detailed understanding of the scope and severity of food insecurity in conflict-affected populations.

Further, as this study was a secondary analysis, it was unclear whether female-headed households were temporarily headed by women, given many of Artsakh’s men were on the frontlines, or if they were permanently female-headed. This distinction is critical as it affects the type and duration of support needed. Temporarily female-headed households may require different forms of assistance, such as temporary financial support, childcare services, and food aid, to cope with the absence of the male household members. In contrast, permanently female-headed households may need more sustained and comprehensive interventions that address long-term economic empowerment, access to education and healthcare, and social support networks. Additionally, the dataset did not include information on the number of children in households or whether there were older individuals present. This omission is significant because both children and older individuals are particularly vulnerable to food insecurity due to their specific nutritional needs and increased susceptibility to the adverse effects of inadequate food access (3, 6). Without this demographic data, our analysis may overlook important factors that contribute to food insecurity and the varying impacts on different household structures. Future studies would benefit from including detailed information on household composition, which could provide a more nuanced understanding of the interplay between displacement and food security.

The methodological strengths of this study include the purposive sampling approach, which ensured representation across different population groups and regions. Additionally, including diverse demographic variables allowed for a detailed analysis of factors influencing food insecurity among displaced populations. However, the study’s focus on refugee and host households in Armenia limits the generalizability of the findings to other conflict-affected settings.

## Conclusion

In summary, this study highlights the profound impact of displacement on food security among populations affected by the 2020 Nagorno-Karabakh conflict. Displaced individuals, particularly those in collective centers, faced significant challenges in securing adequate food, exacerbated by economic vulnerabilities and household composition. These findings underscore the need for targeted humanitarian interventions and policies that address the specific needs of displaced populations to mitigate food insecurity and enhance resilience in conflict-affected regions. Additionally, there is a need for studies that examine the effectiveness of various humanitarian interventions in improving food security among displaced populations.

## Data Availability

Publicly available datasets were analyzed in this study. This data can be found at: https://repository.impact-initiatives.org/document/impact/08a1cbf3/REACH_ARM_Database_MSNA_30122020.xlsx.
